# Impact of the Endocannabinoid System on Bone Formation and Remodeling in p62 KO Mice

**DOI:** 10.3389/fphar.2022.858215

**Published:** 2022-03-22

**Authors:** Christina Keller, Timur Alexander Yorgan, Sebastian Rading, Thorsten Schinke, Meliha Karsak

**Affiliations:** ^1^ Neuronal and Cellular Signal Transduction, Center for Molecular Neurobiology Hamburg (ZMNH), University Medical Center Hamburg-Eppendorf, Hamburg, Germany; ^2^ Department of Osteology and Biomechanics, University Medical Center Hamburg-Eppendorf, Hamburg, Germany

**Keywords:** cannabinoid receptor, p62 (sequestosome 1(SQSTM1), JWH133 (PubChem CID: 6918505), bone, osteoclast (OC), osteoblast (OB), CB2, Paget’s disease of bone (PDB)

## Abstract

Several studies have shown that the G-protein coupled cannabinoid receptor CB2 and its interaction partner p62 are molecularly involved in bone remodeling processes. Pharmacological activation of the CB2 receptor enhanced bone volume in postmenopausal osteoporosis and arthritis models in rodents, whereas knockout or mutation of the p62 protein in aged mice led to Paget’s disease of bone-like conditions. Studies of pharmacological CB2 agonist effects on bone metabolism in p62 KO mice have not been performed to date. Here, we assessed the effect of the CB2-specific agonist JWH133 after a short-term (5 days in 3-month-old mice) or long-term (4 weeks in 6-month-old mice) treatment on structural, dynamic, and cellular bone morphometry obtained by μCT of the femur and histomorphometry of the vertebral bodies in p62 KO mice and their WT littermates *in vivo*. A genotype-independent stimulatory effect of CB2 on bone formation, trabecular number, and trabecular thickness after short-term treatment and on tissue mineral density after long-term treatment was detected, indicating a weak osteoanabolic function of this CB2 agonist. Moreover, after short-term systemic CB2 receptor activation, we found significant differences at the cellular level in the number of osteoblasts and osteoclasts only in p62 KO mice, together with a weak increase in trabecular number and a decrease in trabecular separation. Long-term treatment showed an opposite JWH133 effect on osteoclasts in WT versus p62 KO animals and decreased cortical thickness only in treated p62 KO mice. Our results provide new insights into CB2 receptor signaling *in vivo* and suggest that CB2 agonist activity may be regulated by the presence of its macromolecular binding partner p62.

## Introduction

Bone is a highly dynamic organ that responds to mechanical stress and is constantly remodeled (Ozcivici et al., 2010). Imbalanced activity of osteoblasts (bone-forming cells), osteoclasts (bone-resorbing cells), and osteocytes can cause a variety of skeletal disorders. Diseases of the skeletal system have a high prevalence and great impact on the healthcare system (Gennari et al., 2019). Understanding the mode of action of bone cells and the effects of regulatory molecules and signaling pathways that control these cell types is of particular importance for future treatments.

Osteoblasts, osteoclasts, and osteocytes express the G-protein coupled cannabinoid receptors, CB1 and CB2, with a higher predominance of CB2 receptors (Idris et al., 2005; Ofek et al., 2006). The endocannabinoids anandamide (AEA) and 2-arachidonoylglycerol (2-AG) are produced locally and are degraded by specific enzymes in bone cells (Pertwee, 2015). However, the role of the endocannabinoid system (ECS) in bone remodeling, bone homeostasis, and bone diseases is not fully understood. Mice deficient in CB2 developed an accelerated bone loss with age (Ofek et al., 2006; Sophocleous et al., 2011; Sophocleous et al., 2014a; Sophocleous et al., 2014b). CB2 KO mice on the C57BL/6 background showed decreased trabecular bone volume at the femur and tibia as early as 8 weeks of age (females) (Ofek et al., 2006). This phenotype became even more pronounced in 1-year-old females and males with an osteoporosis-like phenotype with decreased osteoclast number and increased mineral apposition and bone formation rate (Ofek et al., 2006). The age-related osteoporosis in association with increased bone turnover was independently confirmed in the C57BL/6 CB2 KO strain (Sophocleous et al., 2017). However, different mouse lines, gender, and age of the mice contributed to some discrepant results (Ofek et al., 2006; Sophocleous et al., 2014a; Sophocleous et al., 2014b). Thus, the genetic background of the mice was found to influence bone parameters together with CB2 deletion. Studies in 3-month-old females have identified a high bone mass in CB2 KO mice on a CD1 background with increased trabecular bone volume and decreased bone formation rate in the tibia and femur compared with wild-type mice (Sophocleous et al., 2014a). The phenotype in 1-year-old female animals showed a greater loss of trabecular bone volume at the tibial metaphysis, which was associated with a decreased bone formation rate. No genotype-dependent difference was observed in the femur in these old animals. Also, young males showed no difference in the trabecular bone phenotype in CB2 KO animals on the CD1 background (Sophocleous et al., 2014a). Detailed studies to identify molecular explanations for differential findings using gene expression arrays revealed specific differences in gene expression that may contribute to the phenotypes of different CB2 KO mouse strains (Sophocleous et al., 2014b).

Pharmacological blockage of CB1 and CB2 protected mice from ovariectomy-induced bone loss (Sophocleous et al., 2022). CB receptor antagonists primarily mediate inhibition of bone resorption rather than activation of bone formation (Idris et al., 2005). In contrast, CB2 activation had also been described to protect female mice from ovariectomy-induced osteoporosis (Ofek et al., 2006; Sophocleous et al., 2011). It needs to be clarified whether the protective effect of CB2 activation on ovariectomy-induced bone loss is mediated by an inhibitory effect on bone resorption (Rea et al., 2013) or by a stimulatory effect on bone formation (Sophocleous et al., 2011). Stimulation of the CB2 receptor by its selective agonist JWH133 decreased the release of RANK-L and consequently the number and differentiation of osteoclasts, leading to increased mineralization of bone marrow cells from healthy human donors (Rossi et al., 2015). In an *in vivo* model of collagen-induced arthritis (CIA) in mice, loss of trabecular bone parameters, including bone volume, was significantly prevented by JWH133 treatment (Zhu et al., 2019), but osteoclast-mediated osteolysis induced by breast cancer cells was enhanced in a corresponding mouse model (Sophocleous et al., 2015).

To better understand the CB2 receptor signaling pathways, we have previously performed a screen for protein–protein interactions using tandem mass spectrometry. Here, we identified p62 (sequestosome 1, SQSTM1) as an interaction partner for the G-protein coupled CB2 receptor (Sharaf et al., 2019). p62 is a signaling scaffold protein and signaling hub with multiprotein domains that mediate its interactions with various binding partners, implicating the protein in numerous signaling pathways that influence processes such as cell differentiation, survival, osteoclastogenesis, inflammation, obesity, and autophagy (Moscat et al., 2007; Sanchez-Martin and Komatsu, 2018). Binding to the CB2 receptor is mediated *via* the ZZ-type zinc finger (ZZ) domain (Sharaf et al., 2019). The ubiquitin-associated (UBA) domain of the p62 protein clusters mutations identified in patients with familial and sporadic Paget’s disease of bone (PDB) (Morissette et al., 2006; Falchetti et al., 2009), which is characterized by focal and disorganized increases in bone turnover (Roodman and Windle, 2005) and excessive bone-resorbing activity of abnormal osteoclasts (Chamoux et al., 2009). As an autophagy receptor, p62 binds cargo proteins and sequester them to autophagosomes for lysosomal hydrolysis and for the N-degron pathway through the ZZ-domain (Cha-Molstad et al., 2017).

For several years, significant efforts have been made to study p62 in bone diseases (Komatsu et al., 2012; Rea et al., 2013; Sanchez-Martin and Komatsu, 2018). In particular, genetic studies revealed the p.P392L variant of p62 in age-related PDB, leading to increased osteoclastogenic activity (Hiruma et al., 2008). In knockin mouse models of this variant, no histomorphological differences were observed (Hiruma et al., 2008), but Pagetic-like bone lesions were identified (Kurihara et al., 2011; Daroszewska et al., 2018). In mice with a deletion of p62, several results have been published without presenting a clear and congruent bone phenotype (Duran et al., 2004; Zach et al., 2018; Agas et al., 2020). However, it has been predominantly shown that p62 KO mice have an increase in trabecular bone (Zach et al., 2018; Agas et al., 2020). Zach et al. specifically identified an age-dependent phenotype. While 3- and 6-month-old animals showed no changes, older p62 KO mice developed exaggerated bone turnover (a hallmark of PDB) and increased trabecular number along with increased tartrate-resistant acid phosphate (TRAP) activity of distal femur osteoclasts (Zach et al., 2018). The results of the work of Agas et al. (2020) showed an increase in trabecular number and a decrease in trabecular separation as early as two months of age in p62 KO mice (Agas et al., 2020). However, in both studies, the total bone mass of p62 KO mice was similar compared to WT mice (Zach et al., 2018; Agas et al., 2020). In another work, the p62 KO mice did not show any bone phenotype in histomorphometric studies (Duran et al., 2004; Rodriguez et al., 2006). Structural analyses of the long bones of 6- to 8-week-old p62 KO mice revealed normal bone physiology of the tibia and femur. Only after *in vivo* treatment with calciotropic hormone PTHrP, which induces osteoclastogenesis *via* the RANK-L pathway, an increase in osteoclast number was observed in WT but not in p62 KO mice (Duran et al., 2004). Past results argue for the importance of p62 in bone metabolism. Most importantly, p62 is an interaction partner for a number of proteins that play critical roles in bone, such as TRAF 6, RIP1, and aPKC (Sanz et al., 2000; Lamark et al., 2003; Berkamp et al., 2020). It is well known that the binding of p62 to TRAF6 modulates RANK/RANK-L signaling and the NF–κB pathway, thereby regulating osteoclastogenesis (Mcmanus and Roux, 2012) and resulting in increased osteoclastogenesis in p62 KO mice (Duran et al., 2004). Thus, the question arises whether also other interaction proteins act in an altered manner in the absence of p62.

In this work, we explored the possibility that the interaction of CB2 with p62 regulates its function in bone physiology. Therefore, we aimed to investigate whether CB2 activation functions differently in p62 KO mice. We characterized the femur and vertebral bodies of p62 KO and WT mice after short- and long-term *in vivo* activation of CB2 receptors and found slightly different effects of JWH133 on bone homeostasis in p62 KO animals.

## Results

### Increased Trabecular Number in the Femurs of Young p62 KO Mice

**FIGURE 1:**
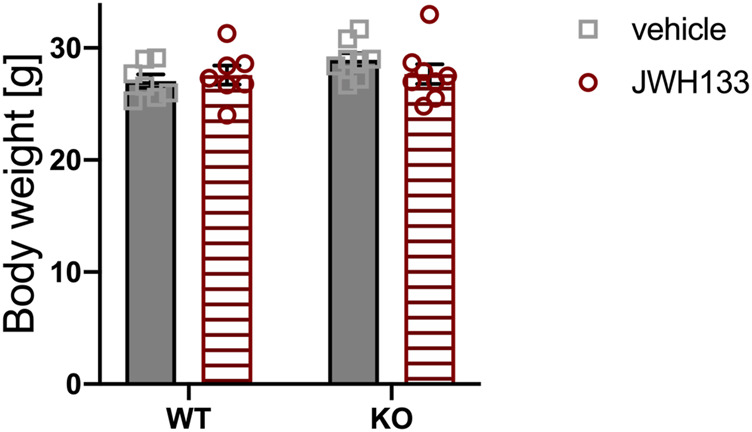
Body weight of p62 KO and WT littermates is not different at the age of 3 months. Male mice were aged 3 months at the beginning of the experiment and were treated for 5 days with vehicle or JWH133. Body weight of mice was similar between all experimental groups. Data were analyzed by using ordinary two-way ANOVA. All error bars show mean ± SEM. Squares and circles represent individual data points. WT vehicle *N* = 7, KO vehicle *N* = 8, WT JWH133 *N* = 8, KO JWH133 *N* = 8.

We hypothesized that the interaction of p62 with CB2 is vital for bone cell differentiation and activation and, therefore, may influence bone remodeling. We combined genetic and pharmacological approaches to investigate the role of the interaction between p62 and CB2 on bone cells and bone remodeling under physiological conditions. For this purpose, 12–13-week-old male mice (WT vehicle *N* = 7, WT JWH133 *N* = 7; KO vehicle *N* = 8, KO JWH133 *N* = 8) were subcutaneously injected with either vehicle or the CB2 agonist JWH133 for a short duration of 5 days. The body weight of the mice was monitored to assess health status and to detect possible effects of treatment. The weight of p62 KO mice was comparable with their WT littermates and was not affected by treatment (Figure 1).

**FIGURE 2:**
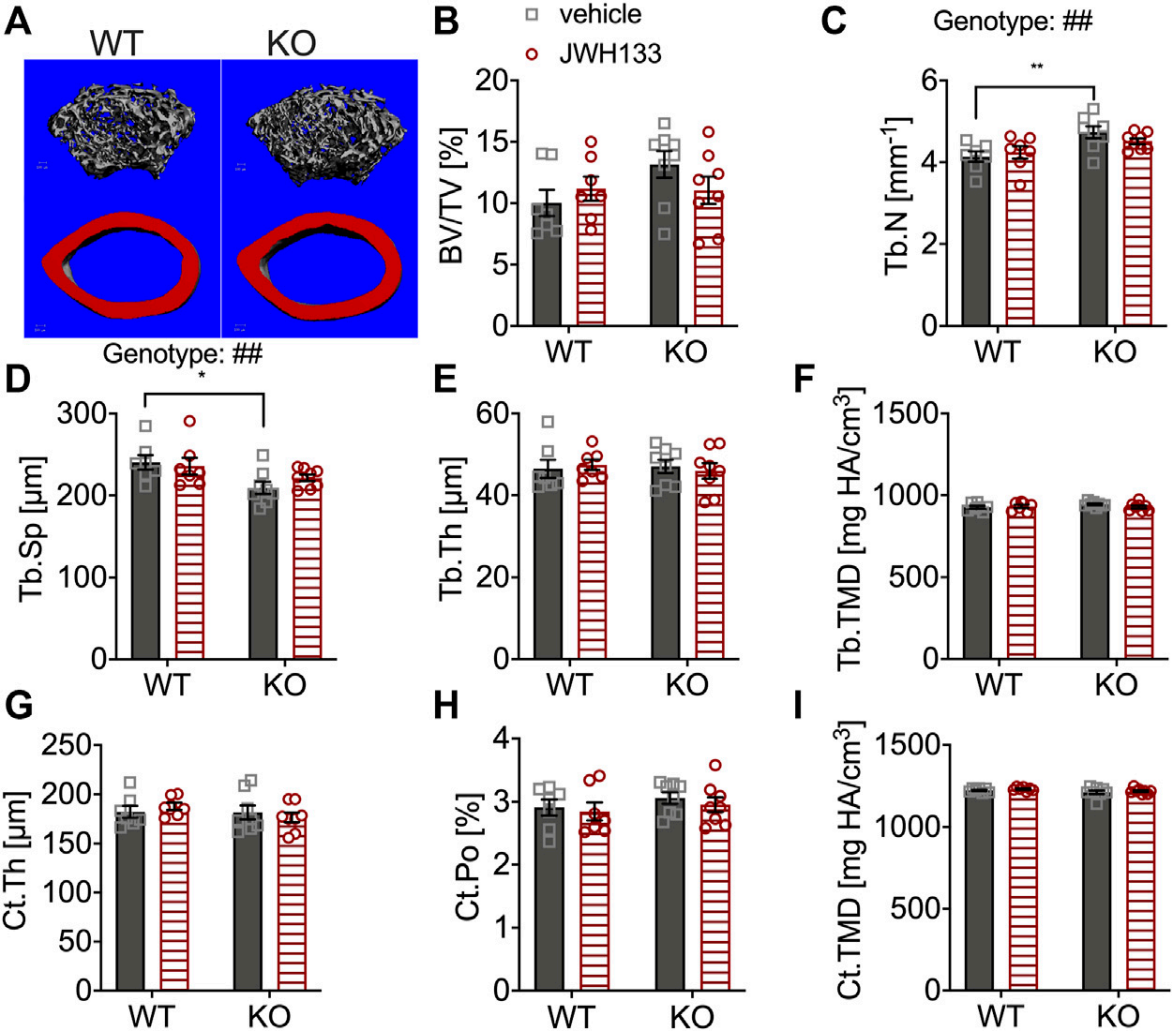
μCT of femurs showed the increased trabecular number and correspondingly reduced spacing in p62 KO mice. **(A)** Trabecular (grey) and cortical (red) bone structure of the femur of WT and p62 KO mice. **(B)** Bone volume per total bone volume (BV/TV) was similar between WT and p62 KO mice and showed no effect of treatment. **(C)** Trabecular number (Tb.N) was significantly increased in vehicle-treated p62 KO mice compared to vehicle-treated WT mice. No effect of genotype was observed after treatment. **(D)** Trabecular spacing (Tb.Sp) was reduced in vehicle-treated p62 KO mice compared to WT mice. **(E)** Trabecular thickness (Tb.Th) showed no difference between p62 KO and WT mice nor an effect of treatment. **(F)** Tissue mineral density (TMD) of trabecular bone was similar between genotypes and showed no effect of treatment. **(G)** Cortical thickness (Ct.Th) of the femur was not influenced by the treatment and showed no difference between genotypes. **(H)** Cortical porosity (Ct. Po) and **(I)** tissue mineral density (TMD) of cortical bone were similar between genotypes and not influenced by the treatment with JWH133. Male mice were aged 3 months by the beginning of the experiment and were treated for 5 days with vehicle or JWH133. Data were analyzed by using ordinary two-way ANOVA and Bonferroni adjusted *p* values, *p < 0.05, **p < 0.01, ***p < 0.001. All error bars show mean ± SEM. Squares and circles represent individual data points. WT vehicle *N* = 7, KO vehicle *N* = 8, WT JWH133 *N* = 8, KO JWH133 *N* = 8.

The distal femoral metaphysis and mid-diaphysis of the mice were measured by μCT to examine bone structure (Figure 2A). Analysis revealed similar trabecular bone volume (BV/TV) in p62 KO and WT mice and no effect of CB2 agonist treatment (Figure 2B). However, the trabecular number (Tb.N) was significantly increased in vehicle-treated p62 KO mice compared with vehicle-treated WT mice (KO vehicle, 4.14 mm^−1^ ± 0.13 mm^−1^, *N* = 8; WT vehicle, 4.74 mm^−1^ ± 0.14 mm^−1^, *N* = 7: *p* = 0.005; Figure 2C) and accordingly resulted in reduced trabecular separation (Tb.Sp) (KO vehicle, 209.31 μm ± 7.28 μm, *N* = 8; WT vehicle, 240.64 m ± 8.89 μm, *N* = 7: *p* = 0.02) (Figure 2D). All other parameters were similar between genotypes and were not affected by treatment with JHW133 (Figures 2E–I). To further investigate the trabecular bone volume, the lumbar vertebrae of the mice were analyzed by structural histomorphometry.

### JWH133 Increased Trabecular Bone Volume in Lumbar Vertebral Bodies With a Stronger Effect in p62 KO Mice

**FIGURE 3:**
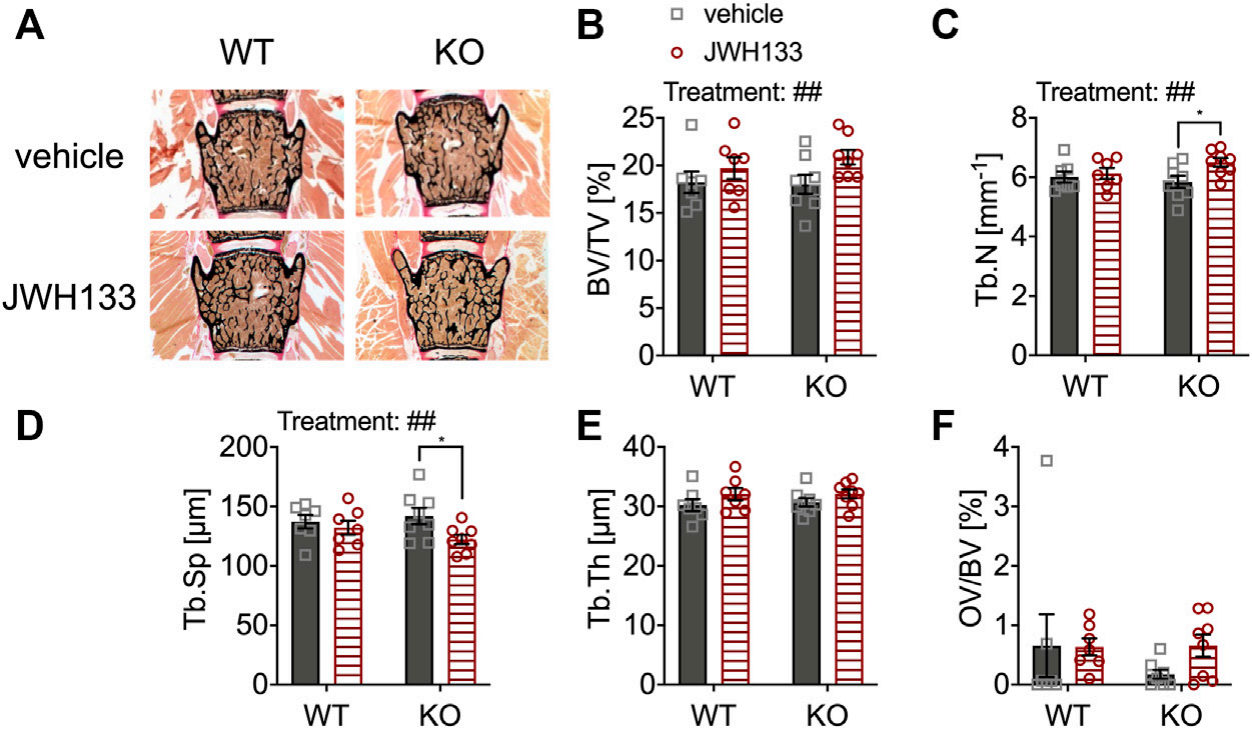
JWH133 increased trabecular bone volume in mice. **(A)** Undecalcified vertebral bodies (L4) stained after von Kossa/van Gieson (black = mineralized bone, red = osteoid) of WT and p62 KO mice that were treated with either vehicle or JWH133. **(B)** Bone volume per tissue volume (BV/TV) was similar between genotypes but was significantly increased by the treatment. **(C)** Trabecular number (Tb.N) was significantly increased by the treatment, and Bonferroni post hoc testing revealed a significant increase in JWH133-treated p62 KO mice compared to vehicle-treated p62 KO mice. **(D)** Trabecular separation (Tb.Sp) was significantly reduced by JWH133, and Bonferroni post hoc testing revealed a significant reduction in JWH133-treated p62 KO mice compared to vehicle-treated p62 KO mice. **(E)** Trabecular thickness (Tb.Th) was similar between genotypes and showed a weak trend of the treatment to increase the thickness. **(F)** Osteoid volume per bone volume (OV/BV) was increased in p62 KO mice after JWH133 treatment but did not reach significance due to high variability. Male mice were aged 3 months by the beginning of the experiment and were treated for 5 days with vehicle or JWH133. Data were analyzed by using ordinary two-way ANOVA and Bonferroni adjusted *p* values, *p < 0.05, **p < 0.01, ***p < 0.001. All error bars show mean ± SEM. Squares and circles represent individual data points. WT vehicle *N* = 7, KO vehicle *N* = 8, WT JWH133 *N* = 8, KO JWH133 *N* = 8.

For histomorphometric evaluation of the bone structure, von Kossa/van Gieson stains of undecalcified spine sections of the same animal groups were examined (vertebral bodies L3 and L4) (Figure 3A). The parameter bone volume per tissue volume (BV/TV) was comparable between genotypes (Figure 3B). However, JWH133 injections for five consecutive days resulted in an increase in bone volume in both WT and p62 KO mice, representing a significant effect of treatment (Figure 3B). This change in total bone mass was caused by an increase in trabecular number and a corresponding decrease in trabecular separation in p62 KO mice (Figures 3C,D). In contrast, trabecular thickness (Tb.Th) was comparable between p62 KO and their WT littermates (Figure 3E). However, a weak trend of the observed treatment effect led to an increase in trabecular thickness in both genotypes (Figure 3E). In contrast to μCT, the histomorphometric analysis includes not only mineralized bone but also osteoid. Osteoid is deposited by osteoblasts and is the portion of bone that is not yet mineralized. Osteoid volume per bone volume (OV/BV) was slightly increased by the treatment in p62 KO mice. However, no significant effect of genotype, treatment, or interaction was detected due to the high variability within the measurement, especially in the WT vehicle group (Figure 3F). These results indicate that increased trabecular bone volume of the lumbar vertebrae of p62 KO mice is primarily due to increased trabecular number after CB2 agonist treatment, suggesting increased osteoblast activity or decreased resorption by osteoclasts as a result of CB2 agonist treatment. To further investigate this effect, we used dynamic and cellular histomorphometry.

### Bone Formation and Mineralization Were Similar Between Genotypes and Not Affected by JWH133

**FIGURE 4:**
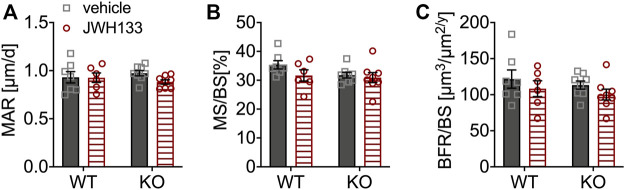
Bone formation and mineralization were similar between genotypes and not affected by JWH133. **(A)** Mineral apposition rate (MAR) of p62 KO and WT mice treated with JWH133 was comparable. **(B)** Mineral surface per bone surface (MS/BS) showed no difference by comparing for genotype and treatment. **(C)** Bone formation rate per bone surface (BRF/BS) showed no effect of treatment and was comparable between genotypes. Male mice were aged 3 months by the beginning of the experiment and were treated for 5 days with vehicle or JWH133. Data were analyzed by using ordinary two-way ANOVA and Bonferroni adjusted *p*nvalues, *p < 0.05, **p < 0.01, ***p < 0.001. All error bars show mean ± SEM. Squares and circles represent individual data points. WT vehicle *N* = 7, KO vehicle *N* = 8, WT JWH133 *N* = 8, KO JWH133 *N* = 8.

Calcein labels were measured in the lumbar vertebrae for a more detailed analysis of bone formation. Mineralization (mineral apposition rate; MAR) of the lumbar vertebrae was not affected by treatment with JWH133, and no differences in mineralizing surface (MS/BS) or bone formation rate (BFR/BS) were observed between p62 KO and WT mice (Figures 4A–C), suggesting normal osteoblast activity. Overall, bone formation and mineralization were not affected by CB2 agonist treatment and were comparable between p62 KO mice and their WT littermates. To further analyze the cause of the increased trabecular bone volume and osteoid deposition after treatment in the mice, cellular histomorphometry was performed.

### JWH133 Treatment Increased the Number and Surface of Osteoblasts and Osteoclasts in Young p62 KO Mice

**FIGURE 5:**
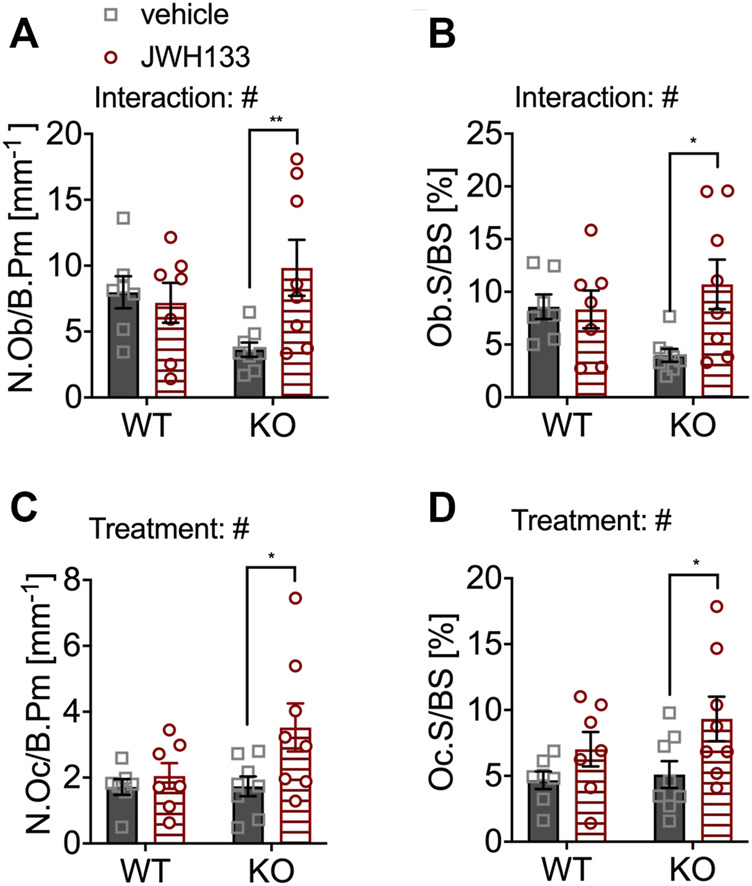
JWH133 treatment increased the number and surface of osteoblasts and osteoclasts in p62 KO mice. **(A)** Number of osteoblasts per bone perimeter (N.Ob/B.Pm) was weakly reduced in p62 KO mice compared to WT mice. The treatment was only effective in p62 KO mice as it increased the number of osteoblasts. **(B)** Osteoblast surface per bone surface (Ob.S/BS) was mildly reduced in p62 KO mice compared to WT mice. The treatment was only effective in p62 KO mice as it increased the surface of osteoblasts. **(C)** Number of osteoclasts per bone perimeter (N.Oc/B.Pm) was similar between genotypes but was significantly increased in p62 KO mice after JWH133 treatment. **(D)** Surface of osteoclasts per bone surface (Oc.S./BS) was similar between genotypes but was significantly increased in p62 KO mice after JWH133 treatment. Male mice were aged 3 months by the beginning of the experiment and were treated for 5 days with vehicle or JWH133. Data were analyzed by using ordinary two-way ANOVA and Bonferroni adjusted *p* values, *p < 0.05, **p < 0.01, ***p < 0.001. All error bars show mean ± SEM. Squares and circles represent individual data points. WT vehicle *N* = 7, KO vehicle *N* = 8, WT JWH133 *N* = 8, KO JWH133 *N* = 8.

The number of osteoblasts was slightly reduced in vehicle-treated p62 KO mice compared with WT control mice (vehicle), but did not reach significance (WT vehicle, 7.99 mm^−1^ ± 1.2 mm^−1^, *N* = 7; KO vehicle 3.63 mm^−1^ ± 0.54 mm^−1^, *N* = 8: *p* = 0.10) (Figure 5A). However, treatment with JWH133 significantly increased the number of osteoblasts in p62 KO mice compared to p62 KO control mice (vehicle) (Figure 5A: KO vehicle, 3.63 mm^−1^ ± 0.54 mm^−1^, *N* = 8; KO JWH133, 9.84 mm^−1^ ± 2.1 mm^−1^, *N* = 8: *p* = 0.01). This increase in osteoblast number was not present in WT mice, resulting in a significant interaction effect (interaction = F_(1.26)_ = 5.6, *p* = 0.03) and a weak trend in treatment (treatment = F_(1.26)_ = 3.3, *p* = 0.08) (Figure 5A). In addition, the percentage of bone surface occupied by osteoblasts was reduced in vehicle-treated p62 KO mice compared with vehicle-treated WT mice but did not reach significance. Treatment of mice with JWH133 significantly increased the percentage of osteoblasts per bone surface in p62 KO mice (Figure 5B: KO vehicle, 4.0 ± 0.6%, *N* = 8; KO JWH133, 10.7 ± 2.4%, *N* = 7: *p* = 0.01), while WT mice were not affected, resulting in a significant effect of interaction (interaction = F_(1.26)_ = 4.5, *p* = 0.04) and trend in the effect of treatment (treatment = F_(1.26)_ = 3.9, *p* = 0.06) (Figure 5B). Next, osteoclasts were analyzed, revealing nearly identical numbers of cells in vehicle-treated p62 KO and WT mice (Figure 5C). Treatment with JWH133 significantly increased the number of osteoclasts in p62 KO mice (Figure 5C: p62 KO vehicle, 1.78 mm^−1^ ± 0.30 mm^−1^, *N* = 8; KO JWH133, 3.58 mm^−1^ ± 0.73 mm^−1^, *N* = : *p* = 0.02) and very slightly in WT mice, still resulting in a significant effect of treatment (Figure 5C). Correspondingly, the percentage of bone surface occupied by osteoclasts was similar in vehicle-treated groups of both genotypes. Again, a statistically significant treatment effect was observed due to an increase in osteoclast surface area in p62 KO mice (p62 KO vehicle, 5.1 ± 1.0%, *N* = 8; KO JWH133, 9.3 ± 1.7%, *N* = 8: *p* = 0.04) and a weak effect was detected in WT mice (Figure 5D: treatment F_(1,26)_ = 6.8, *p* = 0.01: genotype F_(1,26)_ = 1.2, *p* = 0.29: interaction F_(1,26)_ = 0.55, *p* = 0.46).

### Prolonged JWH133 Treatment in Aged Mice Did Not Lead to Detectable Changes in Trabecular and Cortical Bone of the Femur (μCT)

**FIGURE 6:**
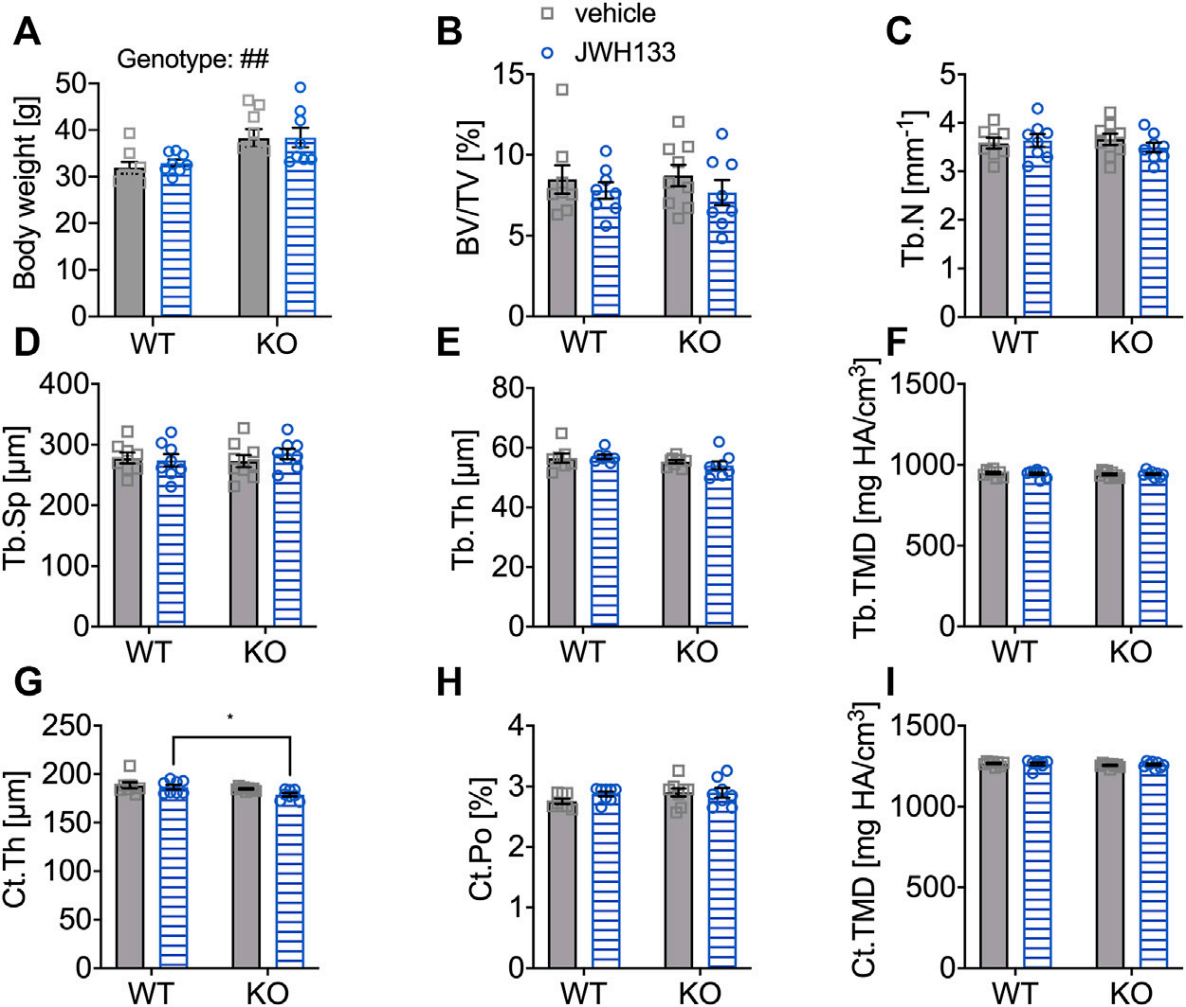
Aged mice did not respond with detectable changes in femoral trabecular and cortical bone (μCT) after a prolonged treatment duration with JWH133. **(A)** Male mice were aged 6 months by the beginning of the experiment. Body weight of p62 KO animals was significantly increased compared to WT mice at the beginning of the experiment. **(B)** Bone volume per tissue volume (BV/TV) was similar between WT and p62 KO mice and showed no effect of treatment. **(C)** Trabecular number (Tb.N) was comparable between genotypes without an effect of treatment. **(D)** Trabecular spacing (Tb.Sp) showed no difference between genotype or treatment. **(E)** Trabecular thickness (Tb.Th) was comparable for genotype and treatment. **(F)** Tissue mineral density (TMD) of the femur was not influenced by the treatment and showed no difference between genotypes. **(G)** Cortical thickness (Ct.Th) was lower in p62 KO mice leading to a significant effect of genotype. **(H)** Cortical porosity (Ct.Po) and **(I)** tissue mineral density (Ct.TMD) of cortical bone were similar between genotypes and not influenced by the treatment with JWH133. Data were analyzed by using ordinary two-way ANOVA and Bonferroni adjusted *p* values, *p < 0.05, **p < 0.01, ***p < 0.001. All error bars show mean ± SEM. Squares and circles represent individual data points. WT vehicle *N* = 8, KO vehicle *N* = 9, WT JWH133 *N* = 8, KO JWH133 *N* = 8.

To test whether prolongation of treatment with the CB2 agonist affects bone mass at a structural level detectable by μCT or structural histomorphometry, a second group of male mice was treated with either vehicle (WT *N* = 8, KO *N* = 9) or JWH133 (WT *N* = 8, KO *N* = 8) for a period of 4 weeks. Body weight of 6-month-old p62 KO mice was significantly increased at the beginning of the experiment compared with their WT littermates (Figure 6A). However, treatment with the CB2 agonist did not affect the body weight of p62 KO and WT mice fed with a standard diet. Moreover, the body weight of the mice remained constant throughout the experiment, indicating good health.

The distal femur of the mice was measured by μCT analysis. The trabecular bone of p62 KO mice and their WT littermates was comparable between genotypes and was not affected by the treatment, as all trabecular bone parameters were comparable (Figures 6B–F). Next, cortical bone was examined and decreased cortical thickness was observed in p62 KO mice compared with WT mice, indicating an effect of genotype that became significant in JWH133-treated mice (Ct.Th, Figure 6G: p62 KO vehicle, 185.0 μm ± 2.0 μm, *N* = 9, WT vehicle, 188.4 μm ± 8.8 μm, *N* = 8: *p* = 0.49; KO JWH133, 179 μm ± 5.1 μm, *N* = 8, WT JWH133, 186.8 μm ± 6.6 μm, *N* = 8: *p* = 0.03). Cortical porosity (Ct.Po, Figure 6H) and cortical tissue mineral density (Ct.TMD, Figure 6I) were comparable between genotypes and were not affected by the treatment.

Taken together, treatment with JWH133 did not induce structural changes in the femur, at least to an extent that could be detected by μCT analysis. In addition, cortical thickness was lower in p62 KO mice compared with their WT littermates at 6 months of age; this difference was not observed in 3-month-old p62 KO mice.

### JWH133 Increased Tissue Mineral Density in the Vertebrae of Aged Mice After a Prolonged Treatment Duration

**FIGURE 7:**
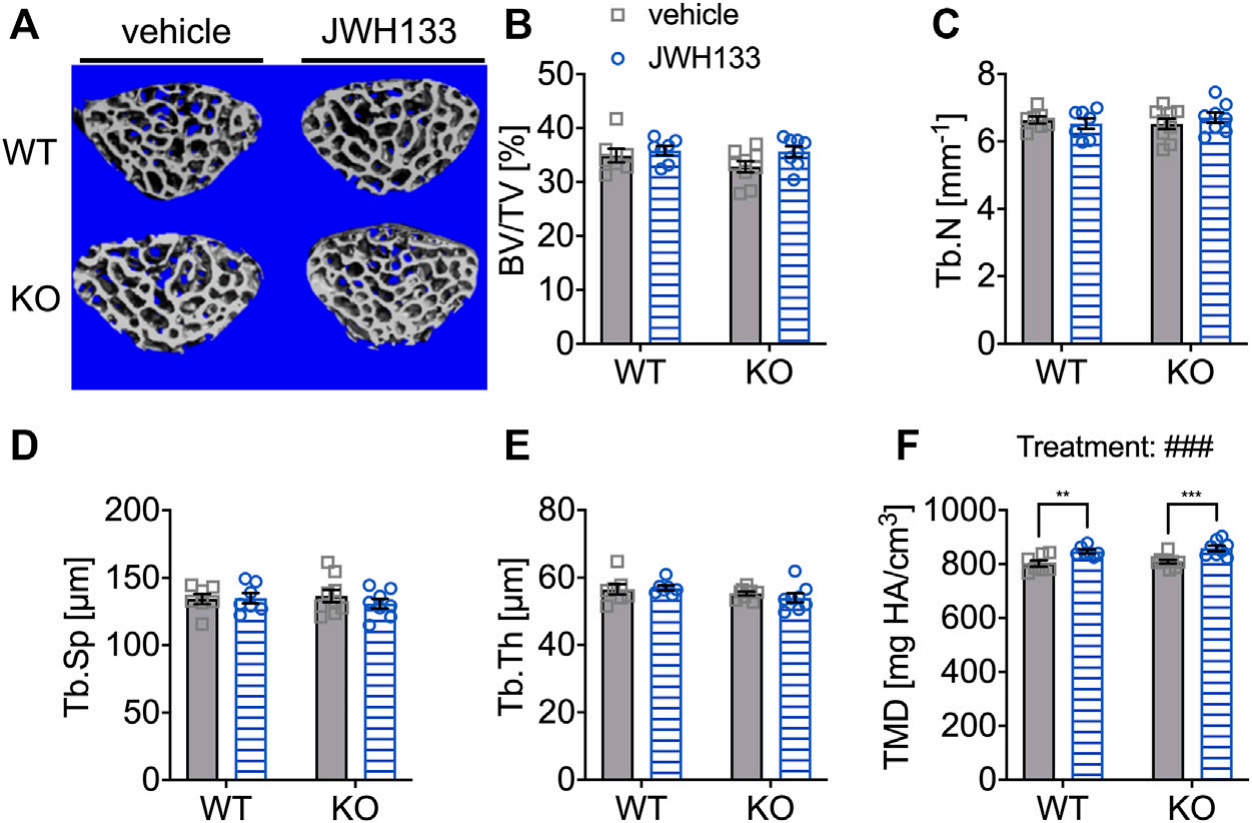
Prolonged treatment with JWH133 increased tissue mineral density in the vertebrae of aged mice. **(A)** Representative image of the vertebral trabecular bone compartment (L5) analyzed by μCT of p62 KO and WT mice treated with vehicle or JWH133. **(B)** Bone volume per tissue volume (BV/TV) was similar between WT and p62 KO mice showed no effect of treatment. **(C)** Trabecular number (Tb.N) was comparable between genotypes showed no effect of treatment. **(D)** Trabecular spacing (Tb.Sp) showed no difference between genotype or treatment. **(E)** Trabecular thickness (Tb.Th) was not different. **(F)** Tissue mineral density (TMD) was significantly influenced by the treatment with JWH133 and affected p62 KO and WT mice. Data were analyzed by using ordinary two-way ANOVA and Bonferroni adjusted *p* values, *p < 0.05, **p < 0.01, ***p < 0.001. All error bars show mean ± SEM. Squares and circles represent individual data points. WT vehicle *N* = 7, KO vehicle *N* = 9, WT JWH133N = 8, KO JWH133 *N* = 8.

Long bones (femurs) and irregular bones (vertebral bodies) are distinct and different bone types (Kappen et al., 2007; Berendsen and Olsen, 2015). The biomechanical loading of these bones differs significantly in mice, with vertebral bodies experiencing less force than the femora. This difference in mechanical stimuli may also lead to altered reactions to factors that can directly or indirectly modulate or influence the response to a stimulus. We expected to find structural changes after the 4 weeks treatment with a CB2 agonist, and as vertebral bodies might respond differently to the treatment, an additional μCT-analysis on the spongy bone of the lumbar vertebral body 5 (L5) was performed. All measured trabecular parameters were analyzed, and no differences were observed between genotypes and treatment groups (Figures 7A–E). Only for tissue mineral density (TMD), JWH133 resulted in a significant increase in both p62 KO and WT mice (Figure 7F: p62 KO vehicle, 809.1 mg HA/cm^3^ ± 21.2 mg HA/cm^3^, *N* = 9, KO JWH133, 859.0 mg HA/cm^3^ ± 28.5 mg HA/cm^3^, *N* = 8: *p* < 0.001; WT vehicle, 802.0 mg HA/cm^3^ ± 30.7 mg HA/cm^3^, *N* = 7, WT JWH133, 847.0 mg HA/cm^3^ ± 18.0 mg HA/cm^3^, *N* = 7: *p* = 0.005).

### Structural Bone Parameters Were Not Affected by Prolonged Treatment With JWH133 in Aged Mice

**FIGURE 8:**
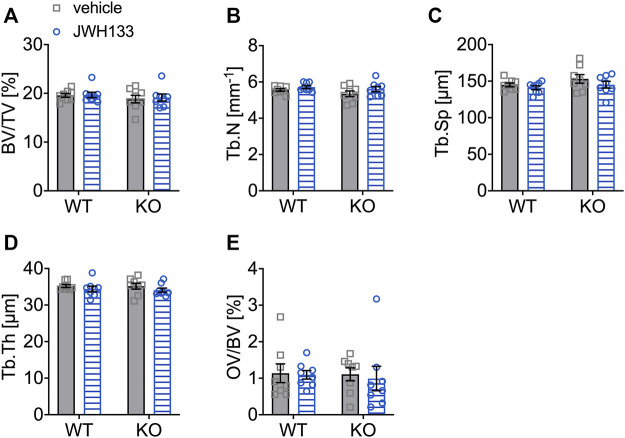
Structural bone parameters were not affected by a 4-week treatment with JWH133 in aged mice. **(A)** Bone volume per tissue volume (BV/TV) was similar between WT and p62 KO mice and showed no effect of treatment. **(B)** Trabecular number (Tb.N) was comparable between genotypes and treatment. **(C)** Trabecular spacing (Tb.Sp) showed no difference between genotype or treatment. **(D)** Trabecular thickness (Tb.Th) was comparable for genotype and treatment. **(E)** Osteoid volume per bone volume (OV/BV) was similar in p62 KO and WT mice and not influenced by the treatment with JWH133. Data were analyzed by using ordinary two-way ANOVA. All error bars show mean ± SEM. Squares and circles represent individual data points. WT vehicle *N* = 8, KO vehicle *N* = 8, WT JWH133 *N* = 8, KO JWH133 *N* = 8.

Since short-term treatment with JWH133 resulted in an increase in osteoid in p62 KO mice, structural histomorphometry was performed on lumbar vertebrae (L1-L4) of the animals after four weeks of treatment with JWH133. Consistent with the μCT data, all structural histomorphometric parameters (Figures 8A–E) were unaffected by treatment and showed no influence of genotype.

### Prolonged Treatment With JWH133 Showed an Opposing Effect on Osteoclasts of 6-Month-Old WT and p62 KO Mice

**FIGURE 9:**
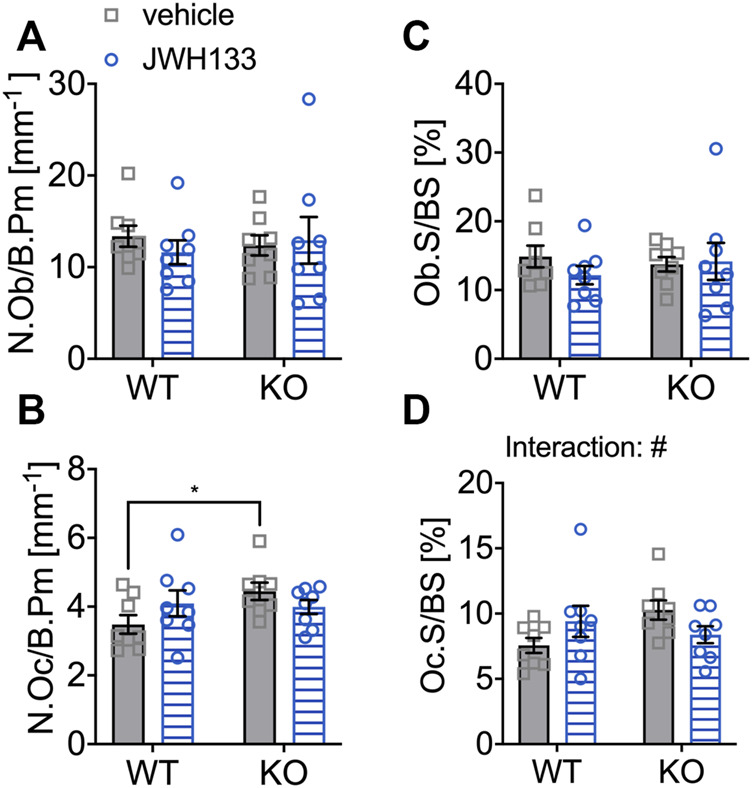
6-month aged WT and p62 KO mice showed an opposing effect on osteoclasts after prolonged treatment with JWH133. **(A)** Number of osteoblasts per bone perimeter (N.Ob/B.Pm) was comparable in vehicle- and JWH133-treated mice and showed no effect of genotype or treatment. **(B)** Number of osteoclasts per bone perimeter (N.Oc/B.Pm) was significantly increased in p62 KO mice (vehicle) compared to WT mice (vehicle). The treatment affected genotypes in an opposite way. **(C)** Osteoblast surface per bone surface (Ob.S/BS) was not altered between genotypes and was not affected by the treatment. **(D)** Osteoclasts per bone surface (Oc.S/BS) were weakly increased in WT mice and reduced in p62 KO mice by the treatment with JWH133 leading to a significant effect of interaction. Data were analyzed by using ordinary two-way ANOVA and Bonferroni adjusted *p* values, *p < 0.05, **p < 0.01, ***p < 0.001. All error bars show mean ± SEM. Squares and circles represent individual data points. WT vehicle *N* = 8, KO vehicle *N* = 8, WT JWH133 *N* = 8, KO JWH133 *N* = 8.

For short-term treatment, an increase in bone cells was detected by treatment with the CB2 agonist only in young p62 KO mice. In the 6-month-old age groups, treated for 4 weeks with the specific CB2 agonist, the number of osteoblasts per bone perimeter (N.Ob/B.Pm) was not affected by treatment and was comparable between genotypes (Figure 9A). The number of osteoclasts (N.Oc/B.Pm) was significantly increased in vehicle-treated p62 KO mice compared to WT control mice (p62 KO vehicle, 4.45 mm^−1^ ± 0.25 mm^−1^, *N* = 8; WT vehicle, 3.48 mm^−1^ ± 0.27 mm^−1^, *N* = 8: *p* = 0.04). Treatment had an opposite effect on genotypes, as the number of osteoclasts was very slightly increased in WT mice (WT vehicle, 3.48 mm^−1^ ± 0.27 mm^−1^, *N* = 8; WT JWH133, 4.10 mm^−1^ ± 0.37 mm^−1^, *N* = 8: *p* = 0.27) and decreased in p62 KO mice (KO vehicle, 4.45 mm^−1^ ± 0.25 mm^−1^, *N* = 8; KO JWH133, 3.99 mm^−1^ ± 0.20 mm^−1^, *N* = 8: *p* = 0.52), still resulting in a trend of an interaction, while no effect of genotype or treatment was observed (Figure 9B). The correlation between the bone cell surface and cell number provides information about the activity status of the cells. The percentage of osteoblasts per bone surface area (Ob.S/BS) was comparable between genotypes and was not affected by treatment (Figure 9C). In osteoclasts, treatment had an opposite effect on genotypes, as the percentage of osteoclasts per bone surface (Oc.S/BS) was weakly increased in WT mice (WT vehicle, 7.56 ± 0.57%, *N* = 8; WT JWH133, 9.41 ± 1.19%, *N* = 8: *p* = 0.27) and slightly decreased in p62 KO mice (KO vehicle, 10.3 ± 0.74%, *N* = 8; KO JWH133, 8.39 ± 0.65%, *N* = 8: *p* = 0.52), resulting in a significant effect of interaction, while no effect of genotype or treatment was observed (Figure 9D).

In summary, 6-month-old p62 KO mice showed a reduced cortical thickness of the femur (μCT) and a tendency toward reduced trabecular bone in the lumbar vertebral body L5 compared with their WT littermates. However, the differences were slight and significant only when the vehicle and JWH133 treatment groups were included. No significant structural bone phenotype was observed in 6-month-old p62 KO mice. Long-term treatment showed an enhancing effect of JWH133 on mineral density in the vertebral body independent of genotype. However, CB2 receptor activation led to an opposing effect between p62 KO and WT mice at the cellular level.

## Discussion

### P62 KO Mice Display a Normal Bone Turnover

General characterization of the bone phenotype of 3-month-old vehicle-treated p62 KO mice revealed an increased trabecular number (Tb.N) in femoral microstructures visualized by μCT. Despite this finding, the overall bone volume was similar between p62 KO and WT mice, suggesting a weak effect of Tb.N on total bone volume in p62 KO mice. Moreover, static histomorphometry of vertebral bodies revealed similar Tb.N and total bone volume in both genotypes, and dynamic and cellular histomorphometry of vertebral bodies showed no differences between p62 KO and WT mice at 3 or 6 months of age, indicating normal bone turnover in p62 KO mice.

The observation that 6-month-old vehicle-treated p62 KO mice had significantly increased numbers of osteoclasts compared with vehicle-treated WT mice did not result in changes in the measured static and dynamic parameters. Because cortical thickness and trabecular bone parameters were similar in vehicle-treated p62 KO and WT mice, this suggests that overall resorption activity was not affected by the loss of p62 at this age. We must consider that the vehicle solution injections may have influenced our obtained data on the overall p62 KO phenotype, but this possibility can be at least partially excluded, as the results are largely consistent with previous publications (Duran et al., 2004; Zach et al., 2018). Thus, our results are consistent with the observations of previous histomorphometric measurements on long bones from another p62 KO mouse line, where a weak but statistically non-significant increase in bone volume, trabecular number, and correspondingly decreased trabecular separation was observed in the tibia and femur of 6- to 8-week-old p62 KO mice (Duran et al., 2004). This result was also confirmed in p62 KO mice at 3 and 6 months of age, which had comparable trabecular numbers to WT mice (Zach et al., 2018). In contrast, a recent publication showed significantly increased trabecular number and correspondingly decreased trabecular separation in μCT of the tibia in young p62 KO mice (8–9 weeks), while total bone volume was again unaffected (Agas et al., 2020).

Aging has a pro-osteoclastogenic effect that is exacerbated by the p62 P394L mutation in a mouse model (Daroszewska et al., 2018). Not only in mice carrying the PDB mutation but also in 15-month-old p62 KO mice, an age-dependent (a hallmark of PDB) exaggerated bone turnover was detected in the distal femora, as indicated by an increased trabecular number accompanied by increased TRAP activity of osteoclasts (Zach et al., 2018). Again, in these aged p62 KO mice, the increase in trabecular number had no effect on total bone mass (Zach et al., 2018). A major limitation of this previous work is that the number of mice studied was relatively small, *N* = 3-4, and thus, the samples analyzed may not be representative. Despite some conflicting results, our observations together with previous publications indicate that the *in vivo* role of p62 in bone physiology is minor and may be age-dependent.

Inconsistencies between studies may be caused by differences between mouse strains and their effects on skeletal microstructure (Papageorgiou et al., 2020). Of note, all studies presented analyzed p62 KO mice on a C57BL/6 background, but used different substrains and different lines (Duran et al., 2004; Zach et al., 2018; Agas et al., 2020). Although genetic differences within the C57BL/6 family are small, there are differences in trabecular indices, bone formation, and bone cell indices (Simon et al., 2013; Sankaran et al., 2017).

### CB2 Agonist JWH133 Had Slight Osteoanabolic Effects in p62 KO Mice

Treatment with JWH133 for 5 days in 3-month-old male p62 KO and WT mice showed a slight effect on bone volume (structural histomorphometry of vertebrae). Otherwise, CB2 activation had almost no effect in healthy WT mice but modulated the bone cell differentiation in p62 KO animals. These results might indicate that p62 as a macromolecular effector and interaction partner influences the function of CB2 receptors. To date, the effects of CB2 signaling in bone have been studied in disease models or estrogen deficiency mimicking postmenopausal osteoporosis (Sophocleous et al., 2022). However, these studies provided evidence that CB2 signaling affects both osteoclast formation and osteogenesis in mice, with some conflicting results (Idris et al., 2005; Ofek et al., 2006; Sophocleous et al., 2011).In an ovariectomy-induced model, the CB2 agonist HU-308 had an osteoanabolic effect and attenuated bone loss in C3H mice (Ofek et al., 2006), which was partly confirmed in C57BL/6 mice using a tenfold lower dose of HU-308 (Sophocleous et al., 2011). Furthermore, CB2 activation in rats reduced bone resorption in a breast cancer–induced (Lozano-Ondoua et al., 2013) and in an osteoarthritis model (Mlost et al., 2021). In a rheumatoid arthritis model, JWH133 suppressed osteoclast formation and differentiation *in vitro* and *in vivo* (Zhu et al., 2019). In contrast, in mice, JWH133 stimulated osteoclast formation (Idris et al., 2008) and enhanced breast cancer cell-induced osteoclastogenesis and osteolysis (Sophocleous et al., 2015).

In the present study, we detected a significant increase in the number of trabeculae and a corresponding decrease in trabecular separation after short-term treatment with JWH133 in the spine of young p62 KO mice only. The amount of osteoid was also slightly increased but showed high variation in p62 KO mice after agonist treatment. These results suggest that the CB2 agonist JWH133 significantly affects bone cell differentiation in young p62 KO mice, resulting in high bone turnover associated with increased osteoid volume and trabecular number. Treatment with JWH133 may lead to a shift in the balance between osteoblast and osteoclast activity. Consistent with this, the number and surface area of osteoblasts and osteoclasts were increased after short-term treatment with JWH133 only in p62 KO. Osteoid deposition may have been responsible for the increase in trabecular bone volume in p62 KO mice after treatment with CB2 agonists. The increase in osteoid may also explain why these changes were not detected by μCT, since μCT only measures the mineralized bone. Mineral apposition rate and bone formation rate were not affected by genotype or treatment, which could be due to the short treatment period before sacrifice, which did not allow sufficient time for the bone to mineralize.

The differential effects of JWH133 treatment in young WT and p62 KO animals might be due to variable CB2 protein levels because p62 is involved in protein degradation as a cargo receptor for autophagy (Pankiv et al., 2007). Assuming that CB2-mediated autophagy depends on the presence and activity of p62, CB2 protein levels might be higher in the absence of p62, and accordingly, a stimulatory CB2 response might be enhanced and promote mineralization in p62 KO mice. Interestingly, a remarkably high expression of CB2 was found in osteoclasts from patients with PDB *in vitro* (Paoletta et al., 2021), so it would also be possible that the strong response in the young p62 KO animals is due to increased CB2 receptor levels. A functional link between CB2 receptor and autophagy has been demonstrated in previous studies showing that CB2 agonists have the potential to promote autophagy (Ke et al., 2016; Wu et al., 2018). Moreover, *in vitro* studies showed that osteogenic differentiation induced by CB2 receptor agonists HU-308 and JWH133 was inhibited when autophagy was blocked (Xu et al., 2020). Further possible explanation for the different responses between p62 KO and WT mice could be altered receptor internalization of CB2 receptors.

Thus, it would be possible that in young p62 WT animals, the CB2 receptors were internalized and, hence, not accessible to the ligand. In contrast, CB2 receptors in young p62 KO mice might be more localized at the plasma membrane, allowing short-term stimulation to produce a stronger effect. Internalization of CB2 receptors is dependent on β-arrestin2, and internalized CB2 receptors colocalized with the early endosome and were recycled to the cell surface after agonist removal (Chen et al., 2014). Recycling of internalized CB2 receptors is assumed to be mediated by proteasome degradation (Chen et al., 2014). Because p62 has been shown not only to be involved in protein degradation but also to interact with β-arrestin2 (Woo et al., 2020), it may be possible that CB2 internalization and recycling are impaired under p62 KO conditions, which needs to be investigated in future work. Of note, JWH133 is significantly biased towards G-protein signaling over β-arrestin coupling and cAMP signaling on the mouse CB2 receptor (Soethoudt et al., 2017).

In a second approach, we aimed to prolong treatment with JWH133 to increase the state of high bone turnover in aged p62 KO mice and induce further changes in bone structure detectable by μCT. An effect on osteoclast number and activity was also observed in these 6-month-old animals after 4 weeks of treatment with JWH133. Here, a slight genotype-dependent effect of CB2 agonist treatment was observed. CB2 activation resulted in a slight decrease in osteoclast number in p62 KO mice and a weak increase in WT littermates. However, this opposite effect of JWH133 on osteoclasts in p62 KO mice compared with WT littermates did not result in differences in bone structure visible on μCT of the femur. In addition, all forms of static and dynamic histomorphometry (vertebral bodies) were similar between p62 KO and WT mice. It is possible that the experimental design was a limitation or that compensatory mechanisms were activated during our *in vivo* experiments. During the short-term treatment, mice were administered daily injections for 5 days. During the long-term treatment experiment, the mice received injections 3 times per week for a period of 4 weeks, so the bioavailability of JWH133 may have been too low, or the receptors may have been desensitized and internalized during the long-term treatment (Udoh et al., 2019; Capote et al., 2021; Patel et al., 2021) and not available for the agonist at the membrane at the right time. Another limiting factor may have been the different ages of the mice used in the short-term and long-term treatment experiments (Willinghamm et al., 2010; Daroszewska et al., 2018). In the older animals, our results could be explained by low CB2 receptor expression, as the number of cannabinoid receptors on bone cells might also be altered at older ages, as has been shown for skeletal muscle (Dalle and Koppo, 2021).

Consistent with our findings that p62 balances CB2 signaling, previous publications have shown that CB2 receptor activation affects the RANK-L (receptor activator of NF-κB ligand) pathway, in which p62 plays an important role (Mcmanus and Roux, 2012). In a mouse model of rheumatoid arthritis, JWH133 suppressed RANK-L-induced IKKα/β phosphorylation, resulting in inhibition of NF-kB signaling activation in WT osteoclasts (Zhu et al., 2019). Of note, this signaling pathway is critical for osteoclastogenesis and inhibition of IKK activation and NF-κB nuclear translocation is impaired in p62 KO animals (Duran et al., 2004). The interplay between CB2 receptor activation and p62 protein levels and their effect on osteoclastogenesis could have direct implications for experimental outcomes. We, therefore, speculate that loss or differential p62 levels in the experimental conditions of previous studies may have contributed to the published paradoxical reports of the effects of CB2 activation on osteoclastogenesis (Bab and Zimmer, 2008; Sophocleous et al., 2015; Zhu et al., 2019).

In conclusion, we hypothesize that the signaling and function of the CB2 receptor are modulated by its interaction with the macromolecular-effector protein p62 *via* influencing the protein levels of this GPCR by either internalization or degradation. Our results demonstrate a molecular link of the endocannabinoid system with p62, as treatment with CB2 receptor agonists resulted in slightly different effects on bone remodeling, bone cell number, and activity in the absence of p62. Although the observed differences are slight, our results suggest an interplay between these two proteins and their signaling complexes. Future studies should investigate whether this molecular link affects bone processes under pathological conditions or at older ages and is thus involved, for example, in disorganized bone turnover or osteoclast activity.

## Materials and Methods

### Drugs

JWH133 (Tocris) was injected s.c. in a concentration of 5 μg/g body weight in a DMSO/Tween 80/NaCl (0.9%) solution (ratio of 1:1:18). Calcein was applied i.p. in a 16 mM NaCl (0.9%)/NaHCO3 buffered solution.

### Mice

Knockout-first p62 mice (C57BL/6N-Sqstm1tm1a (KOMP)Wtsi) were available from the KOMP directory on a C57BL/6N background (ID: 41073) and carried a promoter-driven selection cassette (lacZ and neomycin). In our animal facility, the mice were crossed with C57BL/6J mice (Charles River) for >6 generations to produce fertile offspring that grew normally. Animals were kept in a 12 h:12 h light–dark cycle, with a room temperature of 22°C and 55% humidity and housed with ad libitum access to food and water. All experimental procedures were kept and tested according to the German and European Community laws on the protection of experimental animals and approved by the Behörde für Gesundheit und Verbraucherschutz of the City of Hamburg (project identification code number 139/15 and154/16).

### Short-Term 5-Day Treatment With CB2 Agonist

Two groups of p62 KO and WT littermate mice were used for this experiment. Starting on day 0, mice were injected with calcein (30 mg/kg, ip) and additionally injected with either vehicle or JWH133 (5 mg/kg, s.c.) during days 1–5. Calcein was injected a second time on day 7. Calcein fluorescently labeled newly mineralized bone and determined bone formation rate. All animals were sacrificed on day 9. During the experiment, mice were monitored daily, and the body weight was determined to monitor the health state.

### Long-Term Treatment

Male mice were injected with either JWH133 or vehicle three times per week over a time period of 4 weeks. Vehicle or JWH133 (5 mg/kg) was subcutaneously injected into the 6-month-old animals. Calcein at a concentration of 30 mg/kg was injected intraperitoneally 9 and 2 days prior to the end of the experiment. All animals were sacrificed on day 31 and organs were harvested. Mice were monitored daily, and the body weight was measured before each injection to monitor the health state of mice during the experiment.

### Preparation of Mice for μCT and Histomorphometry

Mice were anesthetized by 80%/20% (v/v) CO_2_/O_2_ inhalation followed by 100% CO_2_ to sacrifice the animals. Skin, fat, and organs were removed. The whole mice (muscle and bone) were fixed in 3,5% PFA for at least 24 h and were then transferred to 80% ethanol until used.

### Microcomputed Tomography (μCT)

This technique is used to image the three-dimensional structure of the cortical and trabecular bone of small rodents. A custom-made sample holder was used to image 12 femurs at the same time (designed by Dr. Timur Yorgan, Institute of Osteology and Biomechanics at the UKE). The right femur of each mouse was used and scanned with a voxel resolution of 10 μm using μCT 40 desktop cone-beam μCT (Scanco Medical, Bruttisellen, Switzerland). Trabecular bone was analyzed in the distal metaphysis with a volume of 2,500 μm–500 μm proximal to the distal growth plate. Cortical bone was also analyzed in a 1 mm long section of the mid-diaphysis. A threshold value of 300 was used for cortical bone evaluation, and a value of 250 was used for trabecular bone.

### Histomorphometry

Static, dynamic, and cellular histomorphometry were already established in the laboratory of Prof. Dr. Michael Amling (UKE, Department of Osteology and Biomechanics). For non-decalcified histology vertebral bodies L1 to L5 were first dehydrated in increasing alcohol concentrations (1–5 h 70% EtOH, two times 1 h 80% EtOH, four times 1 h 96% EtOH, four times 1 h 96% EtOH). Afterward, the samples were incubated for 24 h in an infiltration solution (1,000 ml methyl methacrylate (MMA) destabilized, 3,3g benzoyl peroxide, 100 ml nonylphenol) at 4°C and then transferred to incubation solution II for another 24 h. Next, the samples were embedded in methyl methacrylate and sectioned at 4 μm thickness (for structural and cellular histomorphometry) and 12 μm thickness (dynamic histomorphometry) in the sagittal plane on a Microtec rotation microtome (Techno-Med GmbH, Bielefeld, Germany). 80% isopropyl alcohol and dibutyl ether was applied for stretching. Finally, the slides were dried at 60°C overnight.

### Kossa/van Gieson Staining

Kossa/van Gieson staining was used for structural histomorphometry and to stain mineralized bone matrix black and osteoid red. To remove pMMA from the samples, they were incubated three times in 2-methoxyethylacetate. Afterwards, the slides were rehydrated in descending alcohol concentrations (two times 2 min 100% ethanol, 2 min 96% ethanol, 2 min 80% ethanol, 2 min 70% ethanol, and 2 min 50% ethanol) and rinsed with water. The samples were stained subsequently with 3% silver nitrate and rinsed with water. Then, they were stained in soda-formol solution and rinsed with water. Next, they were stained in 5% sodium thiosulfate and van Gieson solution with interspersed water rinsing steps. The slides were dehydrated in increasing alcohol concentrations and incubated three times in xylene for 5 min. The slides were mounted with DPX mounting solution and covered with a coverslip.

### Toluidine Blue Staining

Additional staining with 1% toluidine blue was used for cellular histomorphometry. Depending on the amount of RNA and DNA within the different tissues and cellular compartments, diverse shades of blue were obtained. The plastic was removed by 2-methoxyethylacetate (incubation for three times and 5 min) and rehydration in descending alcohol concentrations (2 times 2 min 100% ethanol, 2 min 96% ethanol, 2 min 80% ethanol, 2 min 70% ethanol, 2 min 50% ethanol). After rinsing in water, the sections were stained in toluidine blue staining solution for 30 min, followed by water and dehydration through ascending alcohol concentrations. After incubation in xylene for 5 min (three times), the slides were mounted with DPX mounting solution and covered with a coverslip.

### Histomorphometric Quantification

Structural histomorphometry was performed on van Kossa/van Gieson stained slides of the lumbar vertebral. The parameters bone volume per tissue volume (BV/TV), trabecular number (Tb.N), trabecular thickness (Tb.Th), and trabecular spacing (Tb.Sp) were analyzed using Bioquant software. For dynamic histomorphometry, calcein bands were analyzed by the OsteoMeasure histomorphometry system (Osteometrics Inc., United States) on non-stained 12 μm thick lumbar vertebral sections. The mineral apposition rate (MAR), mineral surface per bone surface (MS/BS), and bone formation rate per bone surface (BFR/BS) were determined. Cellular histomorphometry was performed using toluidine blue-stained slides of lumbar vertebral sections. Cellular parameters such as osteoblast surface per bone surface (Ob.S/BS), osteoclast surface per bone surface (Oc.S/BS), number of osteoblasts per bone perimeter (N.Ob/B.Pm), and number of osteoclasts per bone perimeter (N.Oc/B.Pm) were examined using the OsteoMeasure histomorphometry system (Osteometrics Inc., United States).

### Statistics

The two-tailed unpaired t-test was used to make comparisons between WT with p62 KO mice in one variable (*p < 0.05, **p < 0.01, and ***p < 0.001). Two-way repeated measurement ANOVA (two-way ANOVA) was applied to subjects to follow the time course. If the analysis of variance showed a significant effect of genotype, interaction, treatment, or time (#*p* < 0.05, ##*p* < 0.01, and ###*p* < 0.001), then Bonferroni’s multiple comparison post hoc testing was applied (*p < 0.05, **p < 0.01, and ***p < 0.001). The statistical analysis and the graphs were made with GraphPad Prism version 7 (GraphPad Software California, United States). Numerical values are presented as mean ± SEM, and n refers to the number of mice used in this experiment.

## Data Availability

The original contributions presented in the study are included in the article/Supplementary Material, further inquiries can be directed to the corresponding author.
